# Navigating the Long Haul: A Comprehensive Review of Long-COVID Sequelae, Patient Impact, Pathogenesis, and Management

**DOI:** 10.7759/cureus.60176

**Published:** 2024-05-13

**Authors:** Nishant Rathod, Sunil Kumar, Roma Chavhan, Sourya Acharya, Sagar Rathod

**Affiliations:** 1 Medicine, Jawaharlal Nehru Medical College, Datta Meghe Institute of Higher Education and Research, Wardha, IND; 2 Neurosurgery, Trivandrum Medical College, Thiruvananthapuram, IND

**Keywords:** epidemiology, diagnostic criteria, chronic symptoms, multidisciplinary management, pathogenesis, long covid

## Abstract

Long COVID, characterized by persistent symptoms following a SARS-CoV-2 infection, presents a significant public health challenge with wide-ranging implications. This comprehensive review explores the epidemiology, clinical manifestations, pathogenesis, risk factors, diagnosis, patient impact, management strategies, and long-term prognosis of COVID. Despite a varied symptomatology that spans multiple organ systems, including respiratory, neurological, and cardiovascular systems, this condition is primarily associated with chronic inflammation and potential viral persistence. Prevalence varies, influenced by the initial infection severity, demographic factors, and pre-existing conditions. The review emphasizes the necessity for healthcare systems to adapt to the needs of long-COVID patients by developing standardized diagnostic criteria and personalized, multidisciplinary treatment approaches. Current research gaps and future directions are identified, highlighting the urgent need for further studies on pathophysiological mechanisms and effective therapeutic interventions. This review aims to inform healthcare providers, researchers, and policymakers, enhancing patient care and guiding ongoing and future research initiatives. The continuing global focus and collaborative efforts offer hope for improved outcomes for those affected by long COVID, marking an essential step towards addressing this emergent condition comprehensively.

## Introduction and background

Long COVID, also known as post-acute sequelae of COVID-19 (PASC), is a complex medical condition characterized by lingering symptoms that continue or develop after the acute phase of SARS-CoV-2 infection [1]. Typically, these symptoms persist for more than four weeks post-infection, although many patients experience them for several months or longer. Common symptoms include fatigue, shortness of breath, cognitive dysfunction (often referred to as "brain fog"), and a variety of others that affect different organ systems. The definition of long COVID continues to evolve as more research is conducted, highlighting the diverse and multifaceted nature of the condition [2].

Understanding long COVID is critical for several reasons. Firstly, it affects a significant portion of the population, with varying estimates suggesting that a notable percentage of individuals who contract COVID-19 develop long-COVID symptoms [3]. This broad impact calls for a robust health system response to manage healthcare needs. Secondly, the diversity of symptoms and the unpredictability of their progression complicate patient care and require adaptable and personalized treatment approaches. Lastly, COVID poses broader public health challenges, including impacts on workforce participation, healthcare resource allocation, and the mental health of affected individuals. Addressing long COVID effectively is crucial to mitigating its extensive personal and societal consequences [4].

This review aims to comprehensively explore long COVID, encompassing its epidemiology, clinical manifestations, pathogenesis, diagnostic challenges, patient impact, and management strategies. By synthesizing current research and highlighting areas needing further investigation, the review seeks to inform healthcare providers, researchers, and policymakers about effective strategies for addressing long COVID and identify future research and clinical practice directions. The ultimate goal is to enhance the quality of care for patients suffering from long COVID and to contribute to the global understanding of this condition.

## Review

Epidemiology of long COVID

Prevalence Rates

The prevalence of long COVID exhibits considerable variation across different studies and populations. For instance, a study published in the National Center for Biotechnology Information (NCBI) documented a prevalence of 37.3% (95%CI: 30.7%-43.8%) among COVID-19-positive patients in Bhubaneswar [5]. Meanwhile, a population-representative survey in the United States revealed that an estimated 7.3% of all respondents reported experiencing long COVID, corresponding to approximately 18,828,696 adults [6]. A systematic review examining the prevalence of long COVID found that in studies conducted more than 12 weeks post-infection, prevalence rates ranged widely from 0% to 93% [7]. Moreover, a study published in The Lancet highlighted substantial variation in long-COVID prevalence, with rates ranging from 2% in Ghana to 86% in Egypt [8]. Similarly, a study published in the Journal of Clinical Medicine reported a prevalence of 77.7% for long COVID [9]. These disparities in prevalence rates may stem from differences in study populations, methodologies employed, and the specific criteria used to define long COVID. Thus, interpreting and comparing prevalence data necessitates careful consideration of these factors to ensure accuracy and reliability in understanding the burden of long COVID.

Demographic Patterns

Several demographic factors have emerged as significant predictors of long-COVID symptoms. Firstly, gender appears to play a crucial role, with studies consistently indicating a higher likelihood of females experiencing long-COVID symptoms compared to males. A cross-sectional study in Iran demonstrated that females had higher odds of long-COVID symptoms [10]. Similarly, the findings from a population-representative survey in the United States revealed a higher prevalence of COVID-19 among female respondents [11]. Age also exerts a notable influence on the risk of developing long COVID. Research suggests that as age increases, so does the likelihood of experiencing long-COVID symptoms persisting for 4+ and 12+ weeks. There is an observed rise in the reporting of functionally limiting symptoms lasting 4+ and 12+ weeks among older age groups [12]. Additionally, an inverted U-shaped association between long-COVID risk and age was noted, with the highest risk observed in individuals aged 45-54 and 55-69 years [12].

Furthermore, the level of education has been linked to long-COVID susceptibility. The previously mentioned study in Iran found that educated individuals had higher odds of experiencing long-COVID symptoms [10]. Additionally, individuals with underlying health conditions or comorbidities face an elevated risk of developing long COVID. The prevalence of long COVID was notably higher among respondents with comorbidities according to a United States survey [11]. Vaccination status also emerges as a crucial demographic factor associated with long COVID. The survey conducted in the United States revealed that individuals who were not vaccinated or not boosted against COVID-19 exhibited a higher prevalence of long-COVID symptoms than those who were vaccinated or boosted [11]. These findings underscore the intricate interplay between demographic factors and long-COVID susceptibility, highlighting the importance of considering these variables in understanding and addressing the condition's impact.

Variability in Symptoms and Severity

Long COVID, also referred to as PASC, presents as a complex and multidimensional illness affecting a significant proportion of individuals recovering from acute COVID-19 infection [13]. This condition is associated with a diverse range of symptoms and problems, including chronic fatigue, cognitive impairment, respiratory difficulties, cardiovascular irregularities, and psychological distress [13]. However, the precise underlying processes of long COVID remain unknown, presenting significant challenges for healthcare practitioners as it necessitates a multidisciplinary approach to diagnosis, treatment, and rehabilitation [13]. Regarding symptom variability, a study published in PLOS ONE revealed that symptoms of long COVID exhibit fluctuations within individuals over short time scales, displaying heterogeneous patterns of symptom correlation [14]. Additionally, this study identified certain occupations, living status, and smoking habits as risk factors for long-COVID symptoms. A population-representative survey conducted in the United States estimated that 7.3% of all respondents reported experiencing long COVID, equating to approximately 18,828,696 adults [15]. Notably, the prevalence of long COVID was higher among respondents who were female, had comorbidities, or were not vaccinated or boosted against COVID-19. A systematic review examining the prevalence of long COVID found significant variation in prevalence rates, ranging from 0% to 93% in studies conducted more than 12 weeks post-infection [16]. Moreover, the review highlighted substantial between-study heterogeneity and an absence of studies with a low risk of bias, suggesting a gap in the evidence base for robust studies of long-COVID prevalence. These findings underscore the complexity and challenges associated with understanding and addressing long COVID, highlighting the need for further research and evidence-based approaches to manage this condition effectively.

Clinical manifestations of long COVID

Overview of Symptoms

Long COVID, also called PASC, presents as a complex and multifaceted illness affecting a significant proportion of individuals recovering from an acute COVID-19 infection [17]. Among the most common symptoms of long COVID are chronic fatigue, fever, pain, breathlessness, anxiety, chronic fatigue syndrome, and postural orthostatic tachycardia syndrome (POTS) [13,17]. Additional general symptoms may include fatigue, fever, and pain [18]. Notably, the symptoms of long COVID can persist for years, particularly in cases involving new-onset myalgic encephalomyelitis/chronic fatigue syndrome (ME/CFS) and dysautonomia, notably POTS [19]. The pathophysiology of long COVID likely involves long-term organ damage resulting from acute-phase infection, autonomic nervous system damage, immune dysregulation, autoimmunity, endothelial dysfunction, occult viral persistence, and coagulation activation [19]. However, evidence regarding the underlying mechanisms remains limited, with available studies exhibiting heterogeneity [19]. Effectively managing long COVID necessitates a nuanced understanding of its diverse manifestations to address its evolving nature over time [20]. This entails the development of biomarkers for early detection, prognosis, and monitoring, alongside establishing evidence-based guidelines for diagnosis, treatment, and management [20]. Public health initiatives should prioritize increasing awareness, advocating preventative measures, and providing support and services to patients experiencing long-term symptoms [20]. Crucially, collaboration among researchers, healthcare professionals, and patient groups is essential for effectively addressing the challenges posed by long COVID and improving patient outcomes [20]. By fostering interdisciplinary cooperation, stakeholders can work towards enhancing our understanding of long COVID and developing comprehensive strategies to support individuals affected by this condition.

Classification of Symptoms

**Figure 1:**
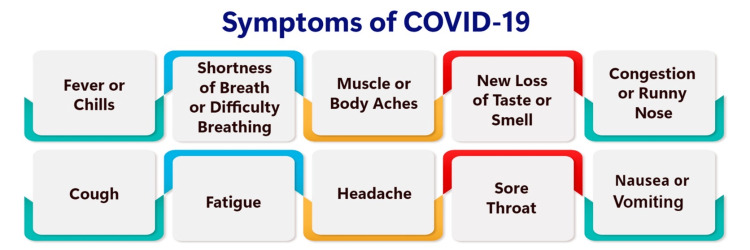
Symptoms of COVID-19 Figure credit: Nishant Rathod

Long-COVID symptoms encompass a range of categories, including neurological, mood disorders, systemic, respiratory, musculoskeletal, ear, nose, and throat, dermatological, and gastrointestinal manifestations [21]. These symptom clusters are identified based on various patient characteristics, such as fatigue status during the acute phase and at the time of presentation, presence of autonomic disorders, and employment status [21]. Among the common symptoms of long COVID are fatigue, fever, pain, breathlessness, anxiety, chronic fatigue syndrome, and POTS [21,22]. It is important to note that these symptoms may evolve, and some individuals with long COVID may receive a diagnosis of POTS following coronavirus infection [23]. Several studies have undertaken the task of classifying long-COVID symptoms into distinct clusters. For example, Tsuchida et al. conducted a cluster analysis of long COVID, categorizing symptoms into three groups: pain, cardiovascular, and the lowest median number of symptoms [21]. Similarly, Ito et al. classified long-COVID symptoms into four clusters based on their severity persisting for 12 months [24]. This systematic classification of symptoms is pivotal for devising effective management and intervention strategies tailored to different sequelae phenotypes in community COVID-19 cases [1]. By enabling healthcare professionals to identify specific symptom clusters, this classification facilitates the administration of precise management and intervention strategies, ultimately enhancing patient care and outcomes [21]. Figure 1 shows the symptoms of COVID-19.

Chronological Evolution of Symptoms

The chronological progression of symptoms in long COVID, also called post-COVID conditions (PCC), needs to be better defined due to its heterogeneity and the limited understanding of its underlying mechanisms [3]. Symptoms can vary from mild to debilitating and may persist for weeks, months, or even years following the initial infection [3]. Among the most commonly reported symptoms of long COVID are fatigue, fever, pain, breathlessness, anxiety, chronic fatigue syndrome, and POTS [3]. Notably, some individuals with long COVID may be diagnosed with POTS after coronavirus infection [3]. The timeline of symptom onset and progression exhibits significant variability among individuals. While some individuals may experience resolution of symptoms within three months after the onset of their initial COVID-19 illness, others may continue to experience symptoms well beyond this timeframe, lasting for years [3]. Moreover, among those with multiple symptoms, some may resolve while others persist [3].

Researchers are still elucidating the long-term prognosis for individuals with long COVID, with the pathophysiology likely involving long-term organ damage stemming from acute-phase infection, autonomic nervous system damage, immune dysregulation, autoimmunity, endothelial dysfunction, occult viral persistence, and coagulation activation [25]. However, evidence regarding the underlying mechanisms remains limited, with available studies exhibiting heterogeneity [25]. Managing long COVID necessitates a nuanced understanding of its diverse manifestations to effectively address its evolving nature over time [13]. This entails the development of biomarkers for early detection, prognosis, and monitoring, as well as the establishment of evidence-based guidelines for diagnosis, treatment, and management [13]. Public health initiatives should prioritize increasing awareness, advocating preventative measures, and providing support and services to patients experiencing long-term symptoms [13]. By fostering comprehensive approaches to management, stakeholders can work towards improving outcomes for individuals affected by long COVID.

Pathogenesis of long COVID

Immune Response and Chronic Inflammation

The relationship between immune response and chronic inflammation in long COVID plays a pivotal role in the persistence of symptoms among affected individuals. Studies have revealed that patients with long COVID often display systemic inflammation and immune dysregulation, characterized by abnormalities in T cell subset distribution and sex-specific perturbations in cytolytic subsets [26]. Additionally, these individuals may exhibit increased frequencies of CD4 T cells primed to migrate to inflamed tissues, exhausted SARS-CoV-2-specific CD8+ T cells, elevated levels of SARS-CoV-2 antibodies, and a miscoordination between their SARS-CoV-2-specific T and B cell responses [26]. Chronic inflammation holds particular significance in long COVID as it offers a potential explanation for the variable nature of the condition's manifestations and provides a framework for exploring treatment options [27]. The dysregulated activation of the immune system, leading to chronic low-grade inflammation, is theorized to contribute to the development and persistence of long COVID [28]. This sustained dysregulation can perpetuate chronic low-grade inflammation, which, in turn, may underlie the persistence of symptoms and the observed multiorgan effects in long COVID [28]. Understanding the interplay between immune response and chronic inflammation is crucial for elucidating the pathophysiology of long COVID and developing targeted therapeutic interventions to mitigate its long-term impact on affected individuals.

Organ-Specific Damage

The relationship between immune response and chronic inflammation in long COVID is crucial for understanding the persistence of symptoms among affected individuals. Studies have consistently shown that patients with long COVID often exhibit systemic inflammation and immune dysregulation, marked by abnormalities in T cell subset distribution and sex-specific perturbations in cytolytic subsets [26]. Furthermore, these individuals may demonstrate heightened frequencies of CD4 T cells primed for migration to inflamed tissues, exhausted SARS-CoV-2-specific CD8 + T cells, elevated levels of SARS-CoV-2 antibodies, and a miscoordination between their SARS-CoV-2-specific T and B cell responses [26].

Chronic inflammation assumes particular significance in long COVID as it offers a potential explanation for the diverse and fluctuating nature of the condition's manifestations. It also provides a framework for investigating treatment avenues [29]. The dysregulated activation of the immune system, leading to chronic low-grade inflammation, is hypothesized to contribute to both the development and persistence of long COVID [30]. This sustained dysregulation may perpetuate chronic low-grade inflammation, which could, in turn, underpin the persistence of symptoms and the observed multiorgan effects associated with long COVID [31]. A comprehensive understanding of the interplay between immune response and chronic inflammation is essential for unraveling the pathophysiology of long COVID and devising targeted therapeutic interventions to alleviate its long-term impact on affected individuals.

Potential Role of Persistent Viral Reservoirs

Persistent viral reservoirs are integral to the pathogenesis of long COVID, influencing the prolonged symptoms observed in individuals recovering from COVID-19. These reservoirs, where the virus can linger in various tissues and organs even after the acute infection has resolved, are implicated in developing chronic symptoms and complications associated with long COVID [32]. Studies indicate that SARS-CoV-2 RNA can persist in diverse body compartments, including the respiratory and gastrointestinal tracts, blood, and other tissues, for extended periods following the acute phase of infection [33]. This persistence of viral RNA and the potential presence of viral proteins can perpetuate ongoing immune responses and inflammation, thereby contributing to the symptoms experienced in long COVID [33]. The existence of viral reservoirs raises pertinent questions regarding the mechanisms underlying the persistence of the virus in specific tissues and its correlation with the onset of long-COVID symptoms [33]. Factors such as the capacity of the virus for replication or non-replication within these reservoirs and the potential for viral shedding and dynamic alterations in viral genome sequences over time are of particular interest in elucidating the pathophysiology of long COVID [32]. By comprehensively understanding the dynamics of viral persistence and its implications for long COVID, researchers can advance insights into the mechanisms driving this condition and potentially identify novel therapeutic targets.

Risk Factors for Developing Long COVID

**Figure 2:**
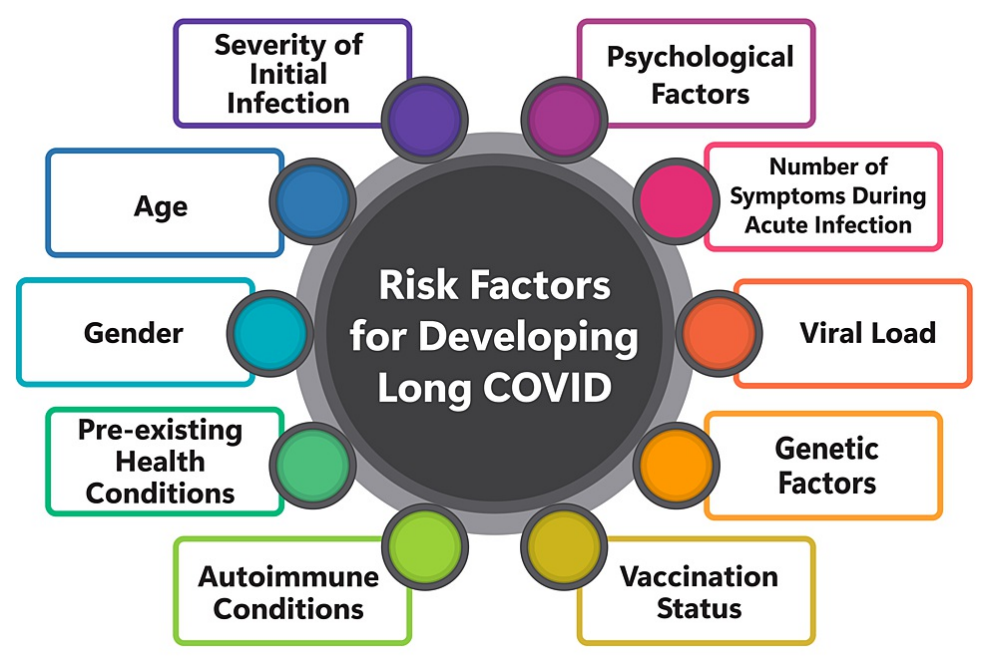
Risk factors for developing long COVID Figure credit: Nishant Rathod

Various risk factors contribute to developing long COVID, encompassing biological sex, age, pre-existing conditions, early symptoms, viral load, autoantibodies, and lifestyle factors [34-37]. Notably, women, mainly those aged 40-60, are at a heightened risk of experiencing persistent symptoms following their initial COVID-19 infection, such as fatigue, breathlessness, cognitive impairment, muscle pain, anxiety, or depression, compared to men [34,35]. Additionally, advancing age is associated with an increased likelihood of developing long COVID, with more than one-fifth (22%) of individuals over 70 reporting symptoms lasting four weeks or longer [34]. Pre-existing conditions, including poor mental health and asthma, are correlated with a greater propensity for reporting long-COVID symptoms [34,35]. Individuals who manifest more than five symptoms during the first week of illness are 3.5 times more likely to develop long COVID than those experiencing fewer symptoms [34]. Moreover, a higher viral load during the early stages of infection and the presence of autoantibodies may elevate the risk of long COVID [34]. Additional risk factors encompass high blood pressure, smoking or former smoking, high BMI, and various comorbidities such as COPD, benign prostatic hyperplasia, and fibromyalgia [35,36]. Understanding and mitigating these risk factors is crucial for identifying individuals at heightened risk of long COVID and implementing preventative measures and targeted interventions to minimize its impact. Figure 2 shows the risk factors for developing long COVID.

Diagnostic approaches for long COVID

Clinical Evaluation

Clinical evaluation for long COVID entails a comprehensive assessment of a broad spectrum of symptoms and signs, given its potential to impact multiple organ systems. The diagnostic approach typically involves an initial broad assessment followed by more focused subsequent evaluations [38,39]. Various tests may be employed during the review, including complete blood count, kidney and liver function tests, thyroid function test, glycated hemoglobin, C-reactive protein, ferritin, WHO functional scale, Wells score, D-dimer, spirometry, chest radiograph, pulse oximetry, 12-lead ECG, 10-minute standing test, cardiac troponin, brain natriuretic peptide, validated cognitive assessment tool, fatigue assessment scale, sub-maximal exercise test, creatinine kinase, and reactivated herpesvirus panel [39].

The cornerstone of managing long COVID revolves around symptom control, appropriate intervention for treatable complications, and detection of new medical diagnoses [39]. While evidence-based pharmacological interventions for preventing and treating COVID are emerging, symptomatic treatment, self-management strategies, and rehabilitation remain pivotal components of clinical care [39]. Diagnostic tools for long COVID are primarily under development, highlighting the need to create and validate biomarkers capable of diagnosing the condition [19]. Biomarker research in ME/CFS may offer valuable insights into long-COVID diagnosis, including electrical impedance blood tests, saliva tests, erythrocyte deformation, sex-specific plasma lipid profiles, and variables related to isocapnic buffering [19]. By advancing biomarker research and diagnostic capabilities, clinicians can enhance their ability to diagnose and manage long COVID accurately, ultimately improving patient outcomes.

Laboratory Investigations

Laboratory investigations are pivotal in supporting, assessing, and diagnosing patients with long COVID. Given the diverse and often overlapping symptoms associated with the condition, relying solely on clinical presentation for diagnosis can be challenging [40]. Furthermore, the absence of well-established evidence-based guidelines for investigating and managing long COVID underscores the importance of targeted laboratory investigations [40]. A critical aspect highlighted in the literature is the necessity for biomarkers to assist in the diagnosis, prediction, and prognosis of long COVID [41]. Biomarkers such as markers of systemic inflammation, cytokines, chemokines, markers of SARS-CoV-2 persistence, and immune dysregulation are under investigation for their potential diagnostic utility in long COVID [41]. The development and validation of these biomarkers are essential for enhancing diagnostic accuracy and guiding treatment decisions for patients with long COVID [41]. Recent research has identified specific proteins and biomarkers in the blood of individuals with long COVID that could serve as diagnostic indicators for the condition [41,42]. These findings offer promising insights into the pathophysiology of long COVID and may facilitate the development of diagnostic tests to aid in identifying individuals experiencing persistent symptoms post-COVID-19 infection [41,42]. By leveraging biomarkers, clinicians can improve their ability to diagnose and manage long COVID effectively, enhancing patient care and outcomes.

Imaging Studies

Imaging studies are crucial in elucidating the pathophysiology of long COVID and identifying potential biomarkers for diagnosis and prognosis. A systematic review examining chest imaging findings in long-COVID patients included 342 individuals who underwent various imaging modalities such as CT, ultrasound scan (USS), chest X-ray (CXR), and MRI [43]. The study population skewed toward males, with a mean age of 59.5 years. CT emerged as the most common imaging modality, utilized in 21.7% of the cohort/case-control study arm and 50% of the case-control study arm. CXR was employed in 55.5% of the cohort study arm, USS in 68.5%, and CT and PET in 70% and 61.5% of the case-control study arms, respectively [43]. Additionally, molecular imaging techniques such as PET and single-photon emission computed tomography (SPECT) have been instrumental in shedding light on the acute and long-term effects of COVID-19 on the human brain. For instance, a retrospective study investigating patients with persistent fatigue more than three weeks after SARS-CoV-2 infection revealed that whole-body PET/CT, including the brain, offers insights into long COVID [44]. Similarly, an observational case-control study focusing on patients with at least one persistent symptom for more than 30 days post-infection demonstrated the utility of PET in detecting neurological abnormalities in long-COVID patients [44]. These imaging studies provide valuable insights into the pathophysiological changes associated with long COVID, aiding in developing diagnostic and prognostic biomarkers while enhancing our understanding of the condition's underlying mechanisms.

Challenges and Limitations

The diagnosis of long COVID presents several challenges and limitations stemming from various factors. Firstly, a lack of standardized diagnostic criteria leads to ambiguity in identifying and defining the condition [45]. This ambiguity is exacerbated by the reliance on self-reporting for symptom identification, which may introduce subjectivity and variability in diagnoses [45]. Furthermore, variations in the definition and prevalence of long COVID across studies and healthcare settings contribute to diagnostic uncertainty [45]. The absence of a robust definition for research and monitoring purposes and overlapping symptomatology with conditions such as ME/CFS complicates the diagnostic process [45,46]. This overlap often necessitates careful differentiation between long COVID and other chronic conditions, posing challenges for healthcare providers. Moreover, the evolving evidence base and the multifaceted impact of long COVID on individuals and society present additional hurdles in accurate diagnosis and management [46]. The scarcity of diagnostic tests during the early stages of the COVID-19 pandemic and variability in inclusion/exclusion criteria in research studies further complicate the diagnostic landscape for long COVID [45]. These challenges underscore the need for standardized diagnostic criteria, improved testing availability, and enhanced collaboration among healthcare professionals to address the complexities of diagnosing long COVID.

Impact of long COVID on patients

Physical Health

Long COVID, also known as PASC, encompasses a complex and multifaceted illness that affects a significant portion of individuals recuperating from an acute COVID-19 infection [19,47]. It manifests through a diverse array of symptoms and challenges, ranging from chronic fatigue and cognitive impairment to respiratory difficulties, cardiovascular irregularities, and psychological distress [19,47]. Despite extensive research, the underlying mechanisms driving COVID remain elusive, posing significant hurdles for healthcare providers regarding diagnosis, treatment, and rehabilitation [19,47]. The physical toll of long COVID is substantial, often marked by persistent fatigue, pain, weakness, and cardiovascular complications [19,47]. These symptoms can endure for extended periods, particularly among specific demographics such as young athletes or those who have experienced severe acute COVID-19 or multisystem inflammatory syndrome in children (MIS-C) [19,47]. Moreover, long COVID can precipitate lasting organ damage, impacting vital systems, including the lungs, heart, nervous system, kidneys, liver, and others [13,19]. In addition to the physical ramifications, COVID can exact a toll on mental health, stemming from experiences of grief, loss, unresolved pain, or persistent fatigue [19]. This complex interplay between physical and mental health challenges further complicates the management of the condition [19]. The cumulative effect of long COVID on the physical and mental well-being of the patients can be debilitating, significantly impacting their daily lives, overall quality of life, and capacity to engage in work activities [19,47]. The repercussions of long COVID extend beyond individual patients, reverberating throughout the broader healthcare system. The strain imposed by long COVID can exacerbate existing workforce shortages and underscore the need for enhanced support structures and workplace protections for healthcare personnel [13,19]. Addressing the multifaceted impact of long COVID necessitates a concerted effort across various sectors to provide comprehensive care and support for those affected by this complex condition.

Mental Health

The mental health ramifications of COVID-19 are profound, as individuals grappling with the condition often contend with symptoms of anxiety, depression, stress, loneliness, and worry [48,49]. These psychological challenges can compound the difficulties associated with managing the physical manifestations of long COVID, further complicating the overall experience for affected individuals [48,49]. Research indicates that individuals with elevated levels of psychological distress before contracting COVID-19 are at a heightened risk of developing long-term symptoms of long COVID [49]. This correlation underscores the intricate interplay between mental health and the propensity for prolonged illness, suggesting that pre-existing psychological factors may influence susceptibility to the condition [49]. Moreover, psychological distress, encompassing conditions such as depression and anxiety, has been linked to an increased vulnerability to long COVID [49]. This susceptibility may stem from the impact of psychological distress on chronic inflammation and immune system dysregulation, potentially heightening individuals' susceptibility to developing the condition [49]. The burden of mental health issues among individuals grappling with long COVID is substantial, permeating various aspects of their daily lives, relationships, and overall well-being [48]. Recognizing the prevalence and severity of these challenges underscores the imperative of addressing mental health concerns and nurturing mental resilience to alleviate the impact of long COVID and enhance outcomes for the affected individuals [48]. Central to this endeavor is ensuring access to comprehensive mental health care tailored to the unique needs of individuals contending with long COVID, particularly those grappling with pre-existing mental health conditions [48]. By prioritizing mental health support as an integral component of long-COVID management, healthcare providers can bolster recovery and promote overall well-being among affected individuals.

Social and Economic Implications

The ramifications of long COVID extend beyond individual health, encompassing significant social and economic implications that reverberate throughout communities and nations [50]. This condition affects a considerable segment of the previously healthy workforce, potentially leading to enduring economic repercussions and strains on healthcare systems [50]. Long COVID's economic impact manifests through direct and indirect costs, encompassing expenses related to healthcare, medications, and social welfare, as well as reduced productivity and diminished tax revenue resulting from individuals exiting the workforce [51]. As the prevalence of COVID persists and its effects become more apparent, its economic burden is expected to escalate, posing challenges to financial stability and societal well-being [50]. Individuals grappling with long COVID face heightened risks of unemployment and financial hardship, exacerbating the overall socioeconomic burden [50]. The diverse nature of long COVID and the nascent state of current research complicate projections of its long-term impacts. Yet, preliminary evidence suggests profound socioeconomic implications, particularly for the most affected individuals [50].

In regions such as the UK and the US, long COVID has contributed to a notable rise in long-term economic inactivity, with a surge in sickness-related absences among the working-age population since the onset of the pandemic [52]. Moreover, labor shortages across various sectors underscore the pervasive impact of long COVID on workforce dynamics, further straining economic stability [51]. The ramifications extend beyond individual spheres, encompassing broader repercussions such as reduced productivity, diminished tax revenue, escalated healthcare costs, and potential disruptions to supply chains [51,53]. Addressing the multifaceted challenges of COVID-19 demands a comprehensive and interdisciplinary approach, integrating healthcare, social support, and economic measures [50]. Key strategies include developing biomarkers for early detection, prognosis, and monitoring alongside evidence-based guidelines for diagnosis, treatment, and management [50]. Public health initiatives should prioritize awareness-raising, advocate preventive measures, and provide robust support and services to individuals grappling with long-term symptoms [50]. Collaborative efforts among researchers, healthcare professionals, and patient advocacy groups are imperative for navigating the complexities of long COVID and enhancing patient outcomes [50].

Quality of Life

The toll of long COVID on quality of life is considerable, surpassing that of some cancers, according to research findings. Individuals grappling with long COVID often contend with profound fatigue, which can rival or exceed the impact of conditions such as cancer-related anemia or severe kidney disease, leading to notably lower health-related quality of life scores compared to individuals contending with advanced cancers [54,55]. The array of symptoms associated with long COVID, including fatigue, breathlessness, anxiety, depression, and cognitive impairment, can severely curtail patients' capacity to participate in daily activities, maintain relationships, and sustain employment, ultimately eroding their overall well-being and quality of life [56]. Moreover, COVID can strain interpersonal relationships, hinder the pursuit of fulfilling life experiences, and exact a toll on mental health, underscoring the profound disruption it inflicts on various facets of patients' lives [56]. Long COVID's economic and social repercussions, particularly its impact on individuals' capacity to engage in gainful employment, further highlight the formidable challenges those living with this condition face. To address these multifaceted needs and enhance the quality of life for the affected individuals, comprehensive support and services are imperative, encompassing healthcare, social assistance, and workplace accommodations [54].

Management strategies for long COVID

Multidisciplinary Approach

The management of long COVID adopts a multidisciplinary framework, which entails a collaborative and integrated strategy involving various healthcare specialties to address the complex and diverse needs of individuals experiencing PASC [57]. This approach acknowledges the multisystemic nature of long COVID, encompassing various organ systems and symptoms, including but not limited to shortness of breath, palpitations, cognitive impairment, and persistent fatigue [57]. Drawing upon the expertise of specialists across diverse disciplines such as neurology, pulmonology, cardiology, infectious diseases, geriatrics, psychology, and hematology, a comprehensive evaluation and management plan can be crafted to effectively address the unique challenges presented by long COVID [58]. The overarching objective of this multidisciplinary approach is to optimize patient care by identifying and managing all potential sequelae of COVID-19, ensuring that individuals receive holistic and coordinated treatment that addresses both clinical and organizational aspects [58]. Such an approach is instrumental in identifying individuals' multifaceted clinical issues and unmet needs with long COVID, facilitating early detection, tailored management strategies, and improved patient outcomes [58].

Symptom-Specific Treatments

The management of long COVID adopts a multidisciplinary framework involving collaboration among various healthcare specialties to address the complex and diverse needs of individuals experiencing PASC [57]. Recognizing the multisystemic nature of long COVID, which encompasses a range of organ systems and symptoms such as shortness of breath, palpitations, cognitive impairment, and persistent fatigue, this approach aims to provide comprehensive care [57]. By leveraging the expertise of specialists in neurology, pulmonology, cardiology, infectious diseases, geriatrics, psychology, and hematology, a tailored evaluation and management plan can be developed to address the unique challenges of long COVID [59]. The primary goal of this multidisciplinary approach is to optimize patient care by identifying and managing all potential sequelae of COVID-19, ensuring that individuals receive holistic and coordinated treatment that considers both clinical and organizational aspects [58]. This collaborative approach is essential for identifying the multifaceted clinical issues and unmet needs of individuals with long COVID, facilitating early detection and personalized management strategies, and ultimately leading to improved patient outcomes [60].

Rehabilitation and Physical Therapy

Rehabilitation and physical therapy are integral components of long-COVID management, aiming to improve symptoms and enhance overall physical function. The Chartered Society of Physiotherapy has established rehabilitation standards for adults aged 18 years and older admitted to hospital with COVID-19, encompassing both acute COVID-19 and long-COVID cases [61]. Physiotherapists play a pivotal role in supporting individuals with long COVID, employing a safety-first approach to address various symptoms and prevent symptom exacerbation [61]. Rehabilitation is deemed essential for individuals living with long COVID, representing one of the core pillars of all long-COVID campaigning efforts [62]. Physiotherapy interventions can target improved joint mobility and pain reduction, particularly beneficial for those experiencing musculoskeletal discomfort associated with long COVID [61]. Physiotherapists also offer crucial education on pacing strategies and energy conservation, empowering individuals to effectively manage their symptoms, avoid overexertion, and optimize daily activities [61]. Moreover, initiatives such as the Long COVID Physio serve as international peer support, education, and advocacy platforms led by physiotherapists living with long COVID and their allies. These organizations contribute to advocacy, policy development, guideline establishment, and research endeavors, with educational resources accessible to individuals living with long COVID and those seeking further understanding [63].

Psychological Support and Counseling

Psychological support and counseling are essential for individuals grappling with long COVID, given its profound psychological implications [64,65]. Psychologists play a pivotal role in guiding patients through their recovery journey and assisting them in preparing for an uncertain future [64]. They offer invaluable support to patients contending with symptoms such as anxiety, depression, cognitive impairment, and trauma [64]. Cognitive Behavioral Therapy (CBT) stands out as a particularly effective form of talking therapy in helping patients effectively manage the chronic symptoms associated with long COVID [66]. Moreover, emotional support from loved ones can significantly alleviate feelings of depression and anxiety experienced by individuals with long COVID [66]. For those with chronic fatigue or impaired mobility, grocery shopping, cooking, and childcare assistance can be immensely beneficial [66]. Online support groups also offer community, providing individuals with long COVID access to support, advice, and additional resources [66]. Psychologists play a crucial role in helping patients and their families establish and reinforce boundaries while addressing issues related to cognitive decline, anxiety, depression, and trauma [64]. Furthermore, involving others and encouraging friends and family members to seek support can help alleviate stress levels within households affected by long COVID [65]. Increasing awareness and understanding of long COVID among healthcare professionals and the general public is paramount in reducing stigma and enhancing support for individuals navigating the complexities of this condition [65].

Long-term prognosis and follow-up

Predictors of Recovery

Predictors of recovery can vary widely depending on the context and the specific population under consideration. Factors influencing long-term recovery in surgical settings encompass bodily and psychological elements [67]. Conversely, in mental health contexts, predictors of recovery may include a range of factors such as sociodemographics, familial risk, early risk factors, premorbid functioning, triggering events, illness-onset characteristics, neurological abnormalities, deficit symptoms, and response to initial treatment [68]. For substance-use disorders, personality traits have been identified as potential predictors of recovery, although their long-term predictive capacity remains poorly understood [69]. Studies focusing on first-episode psychosis have identified various predictors of long-term outcomes, including parental socioeconomic status, family history of schizophrenia spectrum disorders, early developmental delays, childhood adversity, and substance use [68]. Specific indicators such as spontaneous dyskinesia/parkinsonism, neurological soft signs, and educational attainment, particularly the completion of high school, have been associated with symptomatic, functional, and personal outcomes, respectively [68]. In settings such as medium-secure services, interventions targeting dynamic risk factors are essential for enhancing service user outcomes, underscoring the importance of long-term follow-up and personalized interventions [70]. In the intensive care setting, evaluating long-term prognosis and quality of life often involves employing validated assessment methods that consider physical, psychological, and social functioning [71]. These assessments provide valuable insights into the multidimensional aspects of recovery following critical illness and intensive care treatment.

Persistent Symptoms and Complications

Long COVID, also called PASC, manifests as persistent symptoms and complications that can endure for weeks to months following recovery from the acute phase of COVID-19 [72]. A comprehensive systematic review and meta-analysis have identified 55 long-term effects associated with COVID-19, with approximately 80% of infected individuals experiencing one or more persistent symptoms [72]. Among the most prevalent symptoms are fatigue, headache, attention disorders, hair loss, and dyspnea [72]. Significantly, long COVID can affect various organ systems, including respiratory, cardiovascular, renal, gastrointestinal, hepatic, endocrine, and neurological systems [73]. Acute kidney injury (AKI) is a notable complication observed in hospitalized COVID-19 patients, with reported rates ranging widely from approximately 20% to 80%, contingent on the AKI definition employed and its classification [73]. Despite its prevalence, the long-term trajectory of patients with AKI and proteinuria remains poorly elucidated, necessitating further investigation into their natural history and underlying causes [73]. Notably, the incidence of persistent symptoms following hospitalization with COVID-19 remains substantial and stable for up to six months post-discharge [73]. While the majority of COVID-19 cases are mild, approximately 15% of patients develop severe respiratory impairment characterized by diffuse alveolar damage, pulmonary (hyper)inflammatory infiltrates, and microvascular thrombosis, necessitating hospitalization and close medical care [73]. The National Institute for Health and Care Excellence (NICE) defines long COVID as the persistence of signs and symptoms consistent with COVID-19 infection for more than four weeks post-infection, without explanation by an alternative diagnosis [74]. The incidence, natural history, and etiology of long-COVID symptoms remain incomplete and may vary based on viral factors, individual characteristics, and treatment modalities [74]. Increasing evidence underscores a significant proportion of patients experiencing ongoing physical and psychological morbidities post-discharge from SARS-CoV-2 infection, emphasizing the need for continued research and comprehensive patient care [74].

Need for Long-Term Monitoring

Long COVID or PASC encompasses persistent symptoms and complications that endure for weeks to months after recovering from the acute phase of COVID-19 [72]. A thorough systematic review and meta-analysis have identified 55 long-term effects linked to COVID-19, with approximately 80% of individuals experiencing one or more persistent symptoms [72]. Common symptoms include fatigue, headache, attention disorders, hair loss, and dyspnea [72]. COVID-19 can affect multiple organ systems, including the respiratory, cardiovascular, renal, gastrointestinal, hepatic, endocrine, and neurological systems [73]. AKI emerges as a significant complication among hospitalized COVID-19 patients, with reported rates varying widely from approximately 20% to 80%, contingent on the definition and classification of AKI utilized [73]. Despite its prevalence, the long-term trajectory of patients with AKI and proteinuria remains poorly understood, warranting further investigation into their natural history and underlying causes [73]. Moreover, the incidence of persistent symptoms following hospitalization for COVID-19 remains substantial and stable for up to six months post-discharge [73]. Although most COVID-19 cases are mild, approximately 15% of patients develop severe respiratory impairment characterized by diffuse alveolar damage, pulmonary (hyper)inflammatory infiltrates, and microvascular thrombosis, necessitating hospitalization and close medical care [73]. NICE defines long COVID as the persistence of signs and symptoms consistent with COVID-19 infection for more than four weeks post-infection, without explanation by an alternative diagnosis [74]. The incidence, natural history, and etiology of long-COVID symptoms still need to be completed and may vary based on viral factors, individual characteristics, and treatment modalities [74]. Mounting evidence underscores a significant proportion of patients experiencing ongoing physical and psychological morbidities post-discharge from SARS-CoV-2 infection, underscoring the necessity for continued research and comprehensive patient care [74].

## Conclusions

In conclusion, this comprehensive review of long COVID has elucidated the complex nature of the condition, highlighting its wide-ranging and persistent symptoms, diverse pathogenesis, and significant impact on patients' lives. Our findings emphasize the need for a robust response from healthcare providers and researchers. Healthcare professionals must adopt personalized and adaptive management strategies to accommodate the unique challenges presented by long COVID. At the same time, researchers are called upon to investigate its underlying mechanisms further and explore innovative treatments. There is a beacon of hope for patients and families affected by long COVID as the global medical community focuses increasingly on understanding and combating this condition. Continued advancements in research and healthcare practices promise improved patient care and a future where the long-term effects of COVID-19 can be effectively managed, offering patients a better quality of life and optimism for recovery.
